# On-Label or Off-Label? Overcoming Regulatory and Financial Barriers to Bring Repurposed Medicines to Cancer Patients

**DOI:** 10.3389/fphar.2019.01664

**Published:** 2020-01-31

**Authors:** Ciska Verbaanderd, Ilse Rooman, Lydie Meheus, Isabelle Huys

**Affiliations:** ^1^ Department of Pharmaceutical and Pharmacological Sciences, KU Leuven, Leuven, Belgium; ^2^ Anticancer Fund, Strombeek-Bever, Belgium; ^3^ Oncology Research Centre, Vrije Universiteit Brussel, Brussels, Belgium

**Keywords:** drug repurposing, drug repositioning, oncology, regulatory framework, policy, off-label use, incentives

## Abstract

Repurposing of medicines has gained a lot of interest from the research community in recent years as it could offer safe, timely, and affordable new treatment options for cancer patients with high unmet needs. Increasingly, questions arise on how new uses will be translated into clinical practice, especially in case of marketed medicinal products that are out of basic patent or regulatory protection. The aim of this study was to portray the regulatory framework relevant for making repurposed medicines available to cancer patients in Europe and propose specific policy recommendations to address the current regulatory and financial barriers. We outlined two routes relevant to the clinical adoption of a repurposed medicine. First, a new indication can be approved, and thus brought on-label, *via* the marketing authorization procedures established in European and national legislation. Such procedures initiate a detailed and independent assessment of the quality and the benefit-risk balance of a medicinal product in a specific indication, benefiting both prescribers and patients as it reassures them that the scientific evidence is robust. However, the process of marketing authorization for new therapeutic indications entails a high administrative burden and significant costs while the return-on-investment for the pharmaceutical industry is expected to be low or absent for medicines that are out of basic patent and regulatory protection. Moreover, most of the repurposing research is conducted by independent or academic researchers who do not have the expertise or resources to get involved in regulatory procedures. A second option is to prescribe a medicine off-label for the new indication, which is managed at the national level in Europe. While off-label use could provide timely access to treatments for patients with urgent medical needs, it also entails important safety, liability and financial risks for patients, physicians, and society at large. In view of that, we recommend finding solutions to facilitate bringing new uses on-label, for example by developing a collaborative framework between not-for-profit and academic organizations, pharmaceutical industry, health technology assessment bodies, payers, and regulators.

## Introduction

An increasingly popular anticancer treatment development strategy is the clinical investigation of approved and well-characterized non-cancer medicines for new cancer indications, which is known as drug repurposing (Ashburn and Thor, 2004). Drug repurposing is no longer a new concept. It has gained a lot of interest from the research community over the years as it holds the promise of providing safe, timely, and affordable access to new treatment options for patients with unmet medical needs. However, drug repurposing is a broad term covering medicines that are on- or off-patent, shelved or approved, and used “as-is” or reformulated for the new indication (Murteira et al., 2013; Langedijk et al., 2015). In addition, various terms or synonyms are used in scientific literature, such as drug repositioning, drug rediscovery, drug reprofiling, drug rescue and drug redirecting, and a formal regulatory definition does not exist (Langedijk et al., 2015). For the purpose of this review, drug repurposing is confined to the research and development of new anticancer indications for marketed medicinal products that are out of basic patent or regulatory protection.

Key advantages of drug repurposing compared to *de novo* medicine development are the shorter research and development times, the potentially lower development costs and, most importantly, the reduced risk of failure as the safety profile of the medicine is typically well-established (Bertolini et al., 2015; Pushpakom et al., 2019). The opportunities offered by drug repurposing have not gone unnoticed: stories about the success of old “miracle drugs” in new disease areas have been published by the media on numerous occasions (Stone, 2019; 2019). Moreover, several not-for-profit, patient, and governmental organizations have expressed their interest (Hernandez et al., 2017), and the concept has gained recognition among researchers, as illustrated by several influential publications in scientific journals of high impact (Nosengo, 2016; Sachs et al., 2017).

Academia and other research institutes are highly skilled in identifying new candidate molecules for repurposing through computational and experimental screening methods (Oprea et al., 2011; Pushpakom et al., 2019). They also take the lead in *in vitro* and *in vivo* validation of promising repurposing candidates in human tumor models, often in combination with other repurposed or approved anticancer medicines (Würth et al., 2015; Hernandez et al., 2017). As a result, the pipeline with promising candidates for drug repurposing is expanding rapidly (Polamreddy and Gattu, 2019; Serafin et al., 2019; Regulska et al., 2019). With more than 280 compounds, the ReDO_DB list of non-cancer medicines with potential new uses in anticancer treatment is extensive (Pantziarka et al., 2018). About 70 of these medicines are currently being tested in one or more late-stage (i.e., phase II/III, phase III, or phase III/IV) clinical trials for the treatment of cancer patients and more than 95% of those late-stage clinical trials have a non-commercial sponsor.

Success stories of non-cancer medicines being repurposed for anticancer treatment exist. One example is all-trans-retinoic acid (ATRA), a vitamin A derivate used to treat acne that was authorized in Europe to treat adult patients with newly diagnosed low-to-intermediate risk acute promyelocytic leukemia in combination with Trisenox (European Medicines Agency, 2016).Furthermore, drug repurposing is not only gaining momentum in the oncology field, its potential is being recognized in all areas of medicine, including neurology, endocrinology, infectious diseases, cardiology, and psychiatry (Turner et al., 2016; Ferrari and Lüscher, 2016; Fava, 2018; Pantziarka et al., 2018; Parsons, 2019; Farha and Brown, 2019; Miró-Canturri et al., 2019), and especially in those areas with high medical needs such as rare, pediatric, and neglected diseases.

Nevertheless, the substantial increase in scientific knowledge is currently not reflected by equal changes in clinical practice, suggesting that the scientific community is facing challenges to bridge the gap between clinical research and practice (Verbaanderd et al., 2017; Breckenridge and Jacob, 2018). The aim of this study was to portray the regulatory framework relevant for bringing repurposed medicines to cancer patients in Europe based on an analysis of the European and national legislation and guidelines, consultations with experts, and a review of scientific and gray literature in this field. We also discuss specific policy recommendations that have been proposed by various stakeholders to address the current regulatory and financial barriers for clinical adoption of new indications for marketed, off-patent medicines.

## Regulatory Frameworks Relevant for Patient Access to Repurposed Medicines

When developing a new therapeutic indication for an authorized medicine that is out of basic patent and regulatory protection, a key question is how this new use will be translated into clinical practice. Here we outline two options relevant in granting patients access to repurposed medicines. The first option is to approve the new indication and bring it on-label *via* the regulatory procedures established in European and national legislation. The second option is to allow prescription of the new indication off-label based on national frameworks.

### Bringing New Uses On-Label

#### European Regulatory Framework for Marketing Authorization

A marketing authorization application for a new indication initiates a detailed and independent assessment of the quality and the benefit-risk balance of a medicinal product in that specific indication, which is summarized and published in the public assessment report and in the summary of product characteristics (SmPC). Such an assessment benefits both prescribers and patients as it reassures them that the scientific data supporting the new therapeutic indication are robust. Today, the majority of new, innovative medicines are evaluated by the European Medicines Agency (EMA) and authorized *via* the centralized procedure, resulting in a single marketing authorization valid in all EU Member States and countries in the European Economic Area (EEA). However, some products (especially many older medicines) are authorized at national level *via* the decentralized, mutual recognition and national procedures, resulting in national marketing authorizations. Regardless of the route, data requirements and standards for marketing authorization are comparable across the EU.

**Figure 1:**
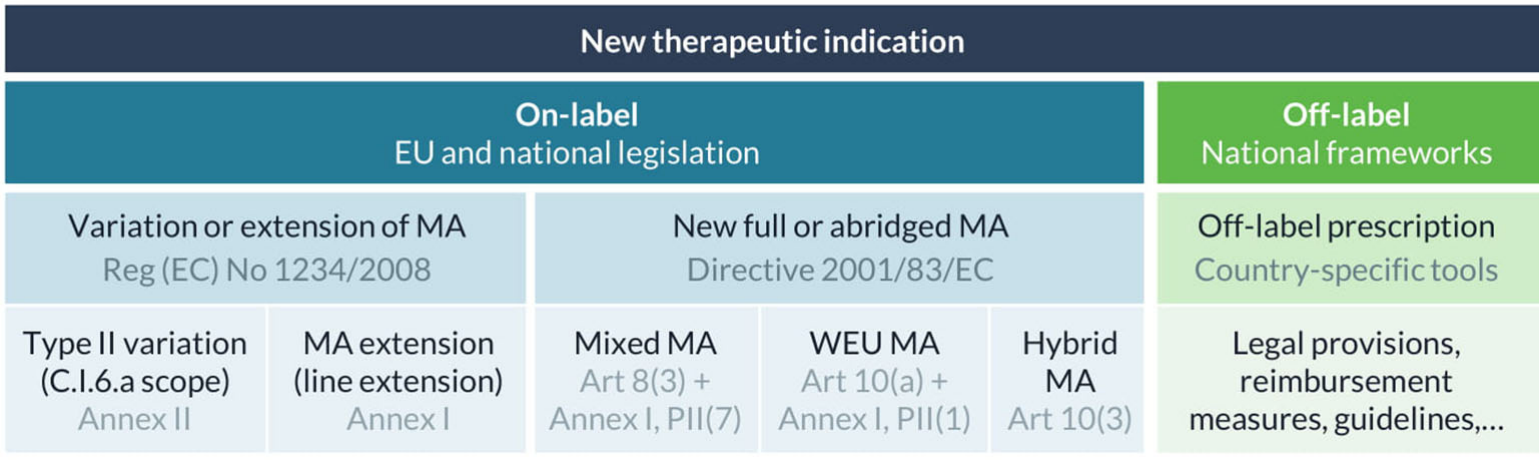
Regulatory frameworks for on- and off-label use of new therapeutic indications in Europe. Marketing authorization (MA), well-established use (WEU), commission regulation (Reg).

A specific European regulatory guideline intended for drug repurposing does not exist but various legal bases are available to get new therapeutic indications approved and bring them on-label (**Figure 1**) (Papakrivos, 2011; Balogh, 2016). First, a marketing authorization holder can apply for a type II variation of its authorized product, more specifically a C.I.6.a scope variation for the addition of a new therapeutic indication or modification of an approved one, under the same marketing authorization. Variations for extension of indication are quite common in oncology and are typically rewarding for a company as this expands the patient population. For example, paclitaxel, initially indicated for the treatment of breast cancer, was later authorized for the treatment of non-small cell lung cancer and metastatic pancreas adenocarcinoma (European Medicines Agency, 2013a; European Medicines Agency, 2015). However, certain changes cannot be granted *via* a variation procedure, for example where a change in indication is accompanied by changes to the strength, pharmaceutical form, or route of administration of the medicinal product (Balogh, 2016; European Medicines Agency, 2019a). In that case, an extension of the marketing authorization (a line extension) needs to be submitted, which will be assessed according to the same procedure as for the initial marketing authorization to which it relates. The extension can either be granted as a new marketing authorization or will be included in the initial marketing authorization (European Commission, 2013). In case the change in therapeutic indication also applies to existing presentations, the application should be presented as a grouping of a line extension and C.I.6.a scope variation (European Medicines Agency, 2019a). All new indications need to be supported by sufficient (pre-) clinical evidence provided by the applicant, and can be supplemented with literature references if available (European Commission, 2013).

In addition, any medicine developer can apply for a new marketing authorization for an existing active substance with a new therapeutic indication. This route would enable a developer to market the product with the repurposed indication under another name, specific for the indication. To make use of scientific data that was published in literature, a full-mixed marketing authorization application can be submitted. This is a stand-alone application in which bibliographical references can be used to support or replace some of the (non-) clinical data in the regulatory dossier. Alternatively, a well-established use or “literature-only” application can be submitted for a well-known active substance if safety and efficacy can be demonstrated by extensive and continued use in the specific indication in the EU over a period of at least 10 years. For this type of application, all test and trial results will be replaced by appropriate scientific literature (with the exception of studies for bridging purposes). For example, mitotane, a well-established medicine that has been used in the treatment of adrenal cortical carcinoma in Europe since 1959, was authorized *via* this pathway in 2004 based on the results of 220 published studies (European Medicines Agency, 2013b).

Another route that is sometimes mentioned in the context of drug repurposing is called the hybrid application route, which is aimed at medicines that differ from their reference medicinal product in therapeutic indication, strength, pharmaceutical form, or route of administration (Vogel, 2012). This abridged procedure allows cross-referencing to existing data in the registration dossier of the reference medicinal product, but also requires new test and trial data to support the new use. However, in practice, this route is mostly used for applications of generic medicines where there are “minor” differences with the reference medicinal product, for instance for minor changes in therapeutic indications within the same therapeutic field (Papakrivos, 2011). This observation is also in line with the guidance provided in Annex II of Chapter 1 of the Notice to applicants Volume 2A about the procedures for marketing authorization (European Commission Health and Food Safety Directorate-General, 2019).

In summary, the legal basis will vary depending on the type of applicant (*e.g.,* only the marketing authorization holder can apply for a variation or extension procedure) and on the amount of data that can be extracted from published literature. Scientific and regulatory advice can be requested at national or European level to better understand the various regulatory pathways and the level of evidence required for marketing authorization of the new indication (Pantziarka, 2017; Shah and Stonier, 2019). Another important aspect that needs to be taken into consideration when selecting which regulatory pathway to follow are the available incentives described below.

#### Regulatory Incentives for Marketing Authorization

**Figure 2:**
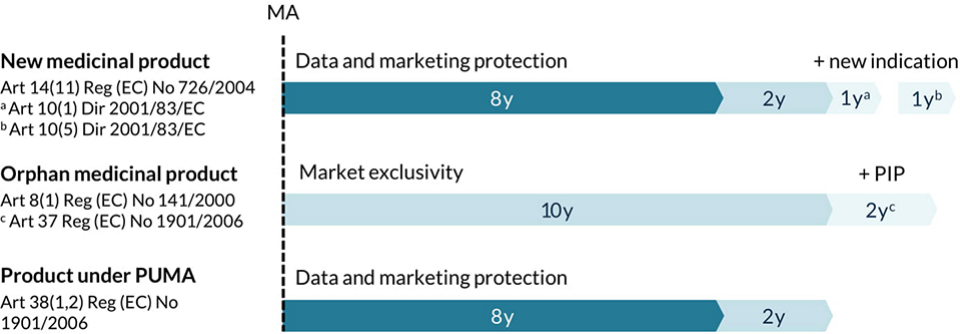
Overview of regulatory exclusivities relevant to the development of new uses in Europe. Marketing authorization (MA), pediatric investigation plan (PIP), pediatric-use marketing authorization (PUMA), regulation (Reg), directive (Dir).

Each new active medicinal product obtains an 8-year period of data protection followed by a 2-year period of marketing protection starting from the date of initial authorization. Any variations and extensions shall be considered as belonging to the same global marketing authorization, in particular with regard to data and marketing protection rules. This means that the 10-year exclusivity period can only be granted once per active substance that is the subject of a marketing authorization held by the same marketing authorization holder. In order to allow companies to recoup investments for the development of new indications for marketed therapies, some additional regulatory exclusivities have been established in Europe (**Figure 2**) (Langedijk et al., 2016; Nayroles et al., 2017). First, the marketing authorization holder can be granted one additional year of marketing protection for one or more new therapeutic indications, with significant clinical beneﬁt in comparison with existing therapies, applied for during the first 8 years (European Commission, 2007a). Second, a non-cumulative period of 1 year of data exclusivity can be granted for a new indication for a well-established substance, if significant pre-clinical or clinical studies were carried out in relation to the new indication (European Commission, 2007b). However, a report from June 2017 on pharmaceutical incentives and rewards in Europe showed that this last incentive had never been granted for any centrally approved substance (Copenhagen Economics, 2018). In certain cases, medicine developers may choose to reformulate, protect (*e.g.,* through second medical use patents) and rebrand an established medicinal product to create sufficient legal and strategic protection from generic competitors (Smith, 2011; Novac, 2013; Dilly and Morris, 2017). For developers other than the original marketing authorization holder, a new full marketing authorization application may even offer a 10-year period of data and marketing protection for the repurposed product (Murteira et al., 2014a).

Repurposing of existing medicines can be particularly useful in areas with high unmet needs, for example to treat patients with rare diseases (Norman, 2013; Murteira et al., 2014a). In fact, studies have shown that about one in five orphan medicinal products are established medicines that were repurposed for a new indication (Davies et al., 2017). Medicinal products that fulfill the criteria for orphan designation [Orphan Regulation (EC) No. 141/2000] are entitled to a 10-year market exclusivity period, possibly followed by two additional years if a pediatric investigation plan (PIP) is completed (**Figure 2**). During this exclusivity period, no other marketing authorization applications can be approved for the same therapeutic indication for a similar medicinal product, including variations or extensions, unless the second medicinal product is safer, more effective, or otherwise clinically superior. Other incentives for orphan medicinal products include protocol assistance, access to the centralized procedure and fee reductions for regulatory procedures. Thalidomide, a medicine that was withdrawn from the market in the 1960s for its disastrous adverse effects upon use during pregnancy, is a well-known example of a medicine that was successfully repurposed for the treatment of multiple myeloma *via* the orphan medicinal product pathway (European Medicines Agency, 2008). Of note, 12 other non-cancer medicines that are currently under investigation for their new use in cancer have obtained an orphan designation in Europe (**Table 1**).

**Table 1:** European orphan designations for non-cancer medicinal products in cancer indications.

Active substance	Original indication	EU OD indication (cancer)	Designation date	Sponsor
Brivudine	Viral infections	Pancreatic cancer	2010	RESprotect GmbH
Chloroquine	Malaria	Glioma	2014	DualTpharma B.V.
Eflornithine + Sulindac	African trypanosomiasis, hirsutism	Familial adenomatous polyposis	2010	Cancer Prevention Pharma Ltd
		Neuroblastoma	2011	Cancer Prevention Pharma Ltd
		Glioma	2016	Orbus Therapeutics Ltd
	Inflammatory conditions	Familial adenomatous polyposis	2013	Cancer Prevention Pharma Ltd
Flucytosine	Fungal infections	Glioma	2018	Richardson Associates Regulatory Affairs Ltd
Itraconazole	Fungal infections	Naevoid basal-cell carcinoma syndrome	2017	Mayne Pharma UK Ltd
Ketoconazole	Fungal infections	Granulosa cell tumors	2017	Grupo Español de Tumores Huérfanos e Infrecuentes (GETHI)
Miltefosine	Leishmaniasis	Cutaneous T-cell lymphoma	2008	ExperGen Drug Development GmbH
Naloxone	Opioid overdose	Cutaneous T-cell lymphoma	2012	Winston Laboratories Ltd
Propranolol	Hypertension	Soft tissue sarcoma	2016	The Anticancer Fund
Valproic acid + Carboplatin	Epilepsy	Diffuse large B-cell lymphoma	2016	Valcuria AB
	Multiple cancer types	Glioma	2018	Dr Ulrich Granzer
Zoledronic acid	Osteoporosis	Glioma	2016	Laboratorio Italiano Biochimico Farmaceutico Lisapharma S.p.A.

Orphan designation (OD), naloxone hydrochloride dehydrate, naloxone.

Source: Community Register of orphan medicinal products for human use, last update in August 2019.

Similar incentives exist for medicines developed exclusively for use in the pediatric population. The EU Pediatric Regulation No. 1901/2006 introduced the Pediatric-Use Marketing Authorization (PUMA) for medicines that have been authorized and can no longer be covered by a supplementary protection certificate (SPC) or a patent (European Commission, 2017). A PUMA offers incentives like automatic access to the centralized procedure, a partial fee exemption and a 10-year period of data and marketing protection (**Figure 2**). Propranolol, a non-selective beta-blocker, is an example of a well-known medicine that was reformulated for use in children with proliferating hemangiomas, authorized *via* the PUMA pathway and successfully rebranded as Hemangiol in the EU (European Medicines Agency, 2014; Léauté-Labrèze et al., 2015). However, to this date (October 2019), only six PUMAs have been granted in the EU, even though scientific literature proposes more than 100 repurposing opportunities in pediatrics (Blatt and Corey, 2013; Rumore, 2016).

#### Pricing, Reimbursement, and Clinical Adoption

The next essential step in bringing a treatment to the patient is setting the price and deciding on the reimbursement of the medicine, which takes place at the national level in the EU and varies across countries (Murteira et al., 2014b; Minghetti et al., 2017). When a new indication is introduced, the pricing of the existing medicine may be re-evaluated and renegotiated in some countries, like France, Italy, and Spain (Nayroles et al., 2017). The introduction of a new indication for a product that was already on the market can lead to price cuts because of a combination of price/volume agreements, external reference pricing, or budget impact analysis (Nayroles et al., 2017). In contrast, a company could ask for an increase in price to compensate for the investments made to develop the new indication, but payers are generally resistant to pay a higher price for a new indication of an existing medicinal product (Shineman et al., 2014; STAMP European Commission expert group, 2017a; Toumi and Rémuzat, 2017).

Furthermore, demonstration of cost-effectiveness is an important factor for reimbursement decisions and for inclusion in national or regional formularies. Cost-effectiveness may be difficult to establish when the price of the repurposed medicine is set significantly higher than the price of the original medicine, especially if effectiveness evidence was available in published literature and no additional testing was required (Simoens et al., 2012; Dooms et al., 2013). For example, chenodeoxycholic acid (CDCA) was a low-cost medicinal product originally developed to treat gallstones in the 1970s and later extensively used off-label to treat patients with the hereditary metabolic disease cerebrotendinous xanthomatosis (CTX). Since 2017, CDCA is officially authorized in Europe as an orphan medicinal product for the treatment of CTX and marketed by Leadiant Biosciences at a much higher price (Van Bossuyt, 2018). This price hike led to the medicine not being reimbursed in several EU countries (Sheldon, 2018).

Ideally, the new indication would also be included in clinical treatment guidelines available at the European level [*e.g.,* European Society for Medical Oncology (ESMO) Clinical Practice Guidelines] and/or the national level to support clinical adoption.

#### Challenges in Bringing New Uses On-Label

Even though repurposing of medicines is considered to be relatively cheap compared to *de novo* medicine development, approval of a new indication can still bring about high costs (STAMP European Commission expert group, 2017a; Breckenridge and Jacob, 2018). These costs include the fees for authorization applications, scientific advice fees, and pharmacovigilance costs for new indications. For example, in 2019, EMA fees for a marketing authorization application start at €291,800, fees for extension of the marketing authorization amount to €87,600, and fees for scientific advice range from €43,700 to €87,600 (European Medicines Agency, 2019b). In addition, applying for marketing authorization of a new indication can place a high administrative burden. The product label and pharmacovigilance system need to be updated for a new indication (STAMP European Commission expert group, 2017a) and, in some cases, a risk management plan or a pediatric investigation plan (PIP) has to be submitted (Nayroles et al., 2017).

Pharmaceutical companies often choose not to invest in new therapeutic indications after expiry of basic patent and regulatory protection periods of their approved products (Langedijk et al., 2016; Nayroles et al., 2017). Patent claims for secondary uses often offer weaker protection compared to the primary basic product patent claims so the risk of free-riding by competitors is high (Sachs et al., 2017). Developing a new indication outside of a company's therapeutic focus is also high-risk and costly (Novac, 2013; Pushpakom et al., 2019). Instead, companies may actually benefit from off-label prescribing of their products because it expands the patient population without them having to apply for a variation or extension of the marketing authorization (Sachs et al., 2017; STAMP European Commission expert group, 2017a). In addition, research has shown that the evidence base supporting new uses for marketed, off-patent medicines in anticancer treatment is largely built through academic or independent research (Pantziarka et al., 2018). A collaboration between academic or independent researchers and the pharmaceutical industry could potentially facilitate pivotal trials and marketing authorization procedures for new indications, but convincing pharmaceutical companies to join forces has proven to be quite challenging (Sukhatme et al., 2014; Polamreddy and Gattu, 2019).

Research foundations or academic institutions are typically not the marketing authorization holder of a repurposed medicine, meaning that they cannot apply for a variation to add a new indication to an existing product label (STAMP European Commission expert group, 2017a). However, there is no legal barrier that prevents them from applying for a new marketing authorization for the medicine in the new indication (Sachs et al., 2017; Davies et al., 2017; Association of Medical Research Charities, 2017). While this approach may theoretically result in more affordable repurposed medicines, several barriers exist. Considering the high costs and administrative burden mentioned before, most non-industry researchers and organizations lack the infrastructure, expertise, and resources to fulfill the necessary requirements for obtaining and maintaining a marketing authorization (including preparation of regulatory dossier, safety monitoring, provision of up-to-date medical information in SmPC, and patient information leaflet, *etc.*) (Weir et al., 2012; Davies et al., 2017; STAMP European Commission expert group, 2017a). In addition, academic researchers are encouraged to disseminate their findings through scientific publications, but they are not (systematically) rewarded for engaging in marketing authorization and market access procedures (Oprea and Mestres, 2012). Current regulatory incentives are tailored to promote development by pharmaceutical companies, while incentives to support further development by the not-for-profit and academic sector are lacking.

### Prescribing New Uses Off-Label

#### National Legislative Frameworks for Off-Label Use

As most repurposed medicines are already authorized and marketed for different indications, they could be prescribed off-label to patients with unfulfilled medical needs in some countries (Devita, 2009; Weda et al., 2017). Off-label use can be defined as any intentional use of an authorized product not covered by the terms of its marketing authorization, for example for another indication, a different patient group, another dose, dose interval, or by another route of administration than indicated in the summary of product characteristics (SmPC) (Vannieuwenhuysen et al., 2015; Weda et al., 2017). Contrary to the strict legal framework for marketing authorization, the actual use of medicinal products in medical practice is not regulated by EU legislation (as confirmed by the European Court of Justice T-452/14 Laboratoires CTRS v Commission, paragraph 79).

Because there is no EU framework for off-label use of medicinal products, Member States manage this in different ways. According to a study commissioned by the European Commission, only 10 out of the 21 Member States that participated in this study have specific policy tools in place to manage off-label use (Weda et al., 2017). For example, France, Hungary, Italy, Greece, and Germany have legal frameworks for off-label use established by their national laws (**Table 2**). These frameworks vary in scope and stringency, and largely focus on the conditions in which off-label use is allowed and potentially also reimbursed. The conditions relate to the scientific basis of the off-label use, the need for explicit informed consent by the patients, the severity of the disease (life threatening or not), or the availability of authorized alternative treatments (Vannieuwenhuysen et al., 2015; Weda et al., 2017). In some countries, clinical guidelines and policies are provided to guide off-label use of medicines [i.e., evidence summaries: unlicensed and off-label medicines (ESUOMs) in the UK (The National Institute for Health and Care Excellence)] and off-label treatment options for which robust scientific evidence exists, are sometimes included in clinical treatment guidelines and formularies (Vannieuwenhuysen et al., 2015; Association of Medical Research Charities, 2017).

**Table 2:** National legal frameworks for off-label use in selected European Union (EU) countries.

Country	Legal provision	Institution	Reimbursement	Legal basis
**France**	Temporary recommendations for use (RTU) scheme	National Agency for Medicines and Health Products Safety (ANSM)	Yes, even if authorized alternative medicinal products exist (for economic reasons)	Art L5121-12-1 and Art R5121-76-1 and following of the Public Health Code
**Hungary**	Individual authorization for off-label prescribing upon request of treating physician	National Institute for Quality and Organizational Development in Healthcare and Medicines (GYEMSZI)	Yes, but on an individual basis within the named patient-based reimbursement system	Section 25 of Act No. XCV of 2005 and Decree No. 44/2004 of the Ministry for Health Care, Social Affairs, and Family
**Italy**	Permissions for off-label use under certain conditions	Italian Medicines Agency (AIFA)	Yes, if included in AIFA “List 648.” Even if authorized alternative medicines exist	Law no. 648/1996, Law no. 94/1998 Art 3(2), Law no. 79/2014
**Greece**	Permissions for off-label use under certain conditions	National Organization for the Provision of Health Services (EOPYY)	Yes, if included in therapeutic protocols and approved by National Healthcare Council (KESY) or upon individual request of healthcare practitioner	Ministerial Decision No. ΔΥΓ3(α)/οικ. ΓΥ/154 and Article 47 of Law 4316/2014
**Germany**	Recommendations for off-label prescribing by four “off-label expert panels”	Federal Joint Committee (G-BA)	Yes, if included in part A of Appendix VI of pharmaceutical directive	Article § 35c(1) of the SGB V

#### Risks of Prescribing New Uses Off-Label

Even though off-label use could offer timely access to new treatment options, it also entails important risks for patients, physicians, and society at large. First, prescribers face significant uncertainty about scientific evidence to support an off-label use because the benefit-risk balance was not assessed by competent authorities and robust clinical data may be lacking (Persidis, 2015; Nayroles et al., 2017). Inconsistencies in off-label use recommendations between treatment guidelines, drug compendia, and evidence reviews further complicate treatment decisions (Abernethy et al., 2009; Green et al., 2016), and so far, only few dedicated data collection systems have been established that could help substantiate the evidence base with real-world data. Another major hurdle for physicians is the risk of legal liability in case a patient experiences any adverse events from using the product outside the approved SmPC (Lenk and Duttge, 2014; Persidis, 2015). Of note, adverse reactions that arise from use of the product outside the terms of the marketing authorization are already captured by the current pharmacovigilance system.

From the patient's perspective, the lack of strong scientific evidence to support off-label use, the lack of information in the patient leaflet and patient materials for the specific indication, the risk of unknown adverse events, and the lack of a risk management plan to protect the patient are key barriers (Persidis, 2015; Eguale et al., 2016). Furthermore, not all countries support the reimbursement of off-label use, so some off-label treatments could be unaffordable to patients (Nayroles et al., 2017). Another challenge is the risk of suddenly losing a medicine that is used extensively off-label for a certain indication, if the manufacturer decides to take it off the market (Verbaanderd et al., 2017). So even though off-label prescribing of repurposed medicines can be very valuable in individual cases, substantial ethical and legal challenges exist (Lenk and Duttge, 2014).

## Policy Recommendations for Clinical Adoption of Repurposed Medicines

The identification of new indications is part of a medicine's life cycle so the more new medicines enter the market, the larger the “toolbox” for repurposing gets. Consequently, the repurposing pipeline is expected to keep growing over time, which is why we need to address the regulatory barriers now. The recommendations below focus on finding solutions to bring new uses on-label because we consider this approach to be more sustainable on the long term, taking into account the aforementioned risks of off-label prescribing. Moreover, previous studies on managing off-label use of medicines have already proposed a wide range of policy options at different levels (Vannieuwenhuysen et al., 2015; Association of Medical Research Charities, 2017; Weda et al., 2017; Dooms and Killick, 2017). The so-called “soft approaches,” such as providing good off-label use practice guidelines, including the new use in existing treatment guidelines and collecting real-world evidence to start knowledge building, were found most relevant to support prescribers and protect patients in the context of off-label use.

### Introducing New Incentives or Removing Current Disincentives

Introducing new incentives may increase industry's willingness to invest in the development of new indications for off-patent medicines. At the EU-level, a first option could be to provide additional or prolonged data and marketing protection periods or improve patent enforceability for second and further medical use claims, however, this could hinder or delay affordable access to medicines (Sachs et al., 2017; Nayroles et al., 2017; Breckenridge and Jacob, 2018; Pushpakom et al., 2019). A second option could be to offer transferable vouchers that grant priority review for future marketing authorization applications, like the US Food and Drug Administration (FDA) priority review vouchers (Muthyala, 2011; Breckenridge and Jacob, 2018). Priority review would theoretically allow a pharmaceutical company to bring another product in their pipeline to the market sooner, but the value of such a voucher is not yet clear. Third, government or philanthropic organizations could award prizes or special research funds to medicine developers, in particular generic producers or small and medium-sized enterprises (SMEs), or independent/academic researchers for developing new therapeutic indications for off-patent medicines (Sachs et al., 2017; Association of Medical Research Charities, 2017; Breckenridge and Jacob, 2018). However, the practical implementation and the value of such prizes or rewards need to be established first.

Alternatively, country-specific incentives may be offered, such as tax incentives [*e.g.,* UK research and development (R&D) credits] or a differential pricing system across indications (Nayroles et al., 2017; Toumi and Rémuzat, 2017; Association of Medical Research Charities, 2017). Differential pricing systems would require the physician to report the indication on their prescriptions for patients, which can be facilitated with e-prescribing software (Roin, 2013). The product could still be prescribed by its generic name for off-patent uses, but also by its brand name for new, patented indications (Duncan and Willoughby, 2016; Breckenridge and Jacob, 2018).

It is uncertain whether the aforementioned incentives would be sufficient to stimulate development of repurposed medicines, and this approach may result in higher medicine prices. A more sustainable approach to encourage the development of new indications for marketed therapies could be to remove or at least reduce the current disincentives for industry, for instance by providing fee reductions or waivers for scientific advice and/or variation applications. Even though this approach would require some additional public investments on the short term, it may offer significant societal benefits.

### Creating a Collaborative European Framework

The gap between the research that is conducted by non-commercial organizations and the need for marketing authorization of a new therapeutic indication cannot be bridged by only addressing the (dis)incentives for the pharmaceutical industry. We need a collaborative, multi-stakeholder, European framework to streamline the identification of repurposing opportunities supported by adequate and robust data and to accelerate the clinical uptake of repurposed medicines that have proven to be safe and effective (STAMP European Commission expert group, 2017b). For that reason, over the past year, the European Commission expert group on Safe and Timely Access to Medicines for Patients (STAMP) has set up a working group to create a visible framework for drug repurposing (STAMP European Commission expert group, 2019). The aim of this framework is to support a not-for-profit or academic stakeholder, termed a “champion,” who has evidence and scientific rationale for a new therapeutic indication that meets particular criteria, in bringing this new indication on-label.

The details of the framework are published elsewhere (Commission Expert Group on Safe and Timely Access to Medicines for Patients (“STAMP”) - European Commission [Internet]) but here we briefly summarize the core components. First, a champion collects sufficient supporting data for a new indication to an off-patent medicine and requests a scientific advice meeting with European or national authorities. Second, the repurposing regulatory scientific advice provides comments and feedback on the presented data package and on the requirements for future data generation (if any). If required, the champion conducts further clinical development in compliance with the scientific advice and/or consolidates the available data. During the development, the champion could seek an immediate or future partnership with one or more marketing authorization holders. Finally, if the data package is considered sufficient, the marketing authorization holder(s) seek(s) an extension, variation, or a new marketing authorization using the existing regulatory pathways (STAMP European Commission expert group, 2019).

In order to test this framework and to assess whether it is able to facilitate an application for a new indication for an off-patent medicine, a pilot project with real-world repurposing cases is expected to start in 2020. However, several outstanding issues already come to mind. First, this framework requires a lot of effort and commitment from the champion, so additional support and incentives for independent or academic researchers to apply for regulatory scientific advice and to collect data intended for regulatory approval are needed (Weir et al., 2012; Kesselheim et al., 2015; STAMP European Commission expert group, 2017a). For instance, a fee waiver or reduction for scientific advice could lower the threshold for independent or academic researchers to seek assistance and would consequently facilitate more efficient data generation (Pantziarka, 2017; Davies et al., 2017). Second, most researchers have no or very limited experience with preparing a scientific advice briefing document, so additional guidance documents and better education on regulatory procedures may be useful (STAMP European Commission expert group, 2017a). The latter could be addressed by the Horizon 2020 coordination and support action on “strengthening regulatory sciences and supporting regulatory scientific advice” that is currently in preparation. Finally, the outcome of this framework is uncertain as it fully depends on the willingness of the marketing authorization holder(s) to apply for a variation, extension, or new marketing authorization. Nonetheless, this type of EU-wide framework to facilitate drug repurposing is definitely encouraging for involved researchers and any outstanding issues can be identified and addressed during the pilot project.

### Initiating Legislative Changes in Europe

Some stakeholders are convinced that legislative changes will be needed. One proposal is to introduce a legal provision in EU legislation that allows third parties, such as research institutes or foundations, to apply directly for a variation or extension of marketed products (Giovannoni et al., 2015; STAMP European Commission expert group, 2017a). However, the implementation of this approach would require a lot of time and would initiate complicated discussions about the responsibility and legal liability for the marketing authorization. Another idea is to strengthen the role of the regulatory agencies in bringing new uses on-label by requiring them to follow-up on any evidence made available to support new indications for approved medicines. Regulators could encourage (or even enforce) the need for a variation application by marketing authorization holders based on positive results from clinical trials conducted by third parties. During the STAMP expert meetings, Articles 23 (Murteira et al., 2013), 31 and Annex I (Oprea et al., 2011; 2019) of Directive 2001/83/EC and Article 16 (Murteira et al., 2013) of Regulation (EC) No 726/2004 were mentioned as potential legal bases for this type of “efficacy vigilance” (STAMP European Commission expert group, 2017a). Yet, it soon became clear that this approach would not be sustainable because it raises uncertainties about the responsibility for the evidence and it entails increased legal liability for regulatory agencies. An alternative approach would be for the executive director of the EMA or the Commission representative to request non-binding (“soft”) recommendations or advice of the Committee for Medicinal Products for Human Use (CHMP) on all new uses [Article 5 (3) of Regulation (EC) No 726/2004]. However, a scientific opinion on a new therapeutic indication by EMA that is not taken up in the marketing authorization may promote off-label use, which is not in line with their policy.

Legislative actions relevant to drug repurposing have already been proposed in some countries but with limited success so far. In the UK, the Off-Patent Drugs Bill was launched to facilitate drug repurposing by requiring the Secretary of State to seek licenses for off-patent medicines in new indications and to request technology appraisals for these medicines from the National Institute for Health and Care Excellence (NICE) but this bill failed in Parliament in 2015 (House of Commons 2015, 2015). In the US, a bill was introduced in the senate in September 2018, called the “Making Objective Drug Evidence Revisions for New Labeling Act” or “MODERN Labeling Act,” which could be relevant to drug repurposing (115th Congress (2017–2018), 2018). The not-for-profit organization Friends of Cancer Research started the initiative to address outdated generic product labels in the US (Friends of Cancer Research, 2018; Shea et al., 2018). The proposed bill aims to establish a process for the FDA to determine whether the labeling of generic medicines needs modifying, including medicines with relevant accepted uses in clinical practice (supported by evidence that could meet the standards for approval) that are not reflected in the approved labeling.

## Conclusion

Drug repurposing holds the promise of accelerating access to safe and affordable treatment options, especially for patients with rare, pediatric, or neglected diseases. The goal is not to replace *de novo* medicine development but rather to complement it. Even though various regulatory pathways already exist to make repurposed medicines available to cancer patients, important challenges occur in bringing new uses on-label (Simsek et al., 2018). To address current challenges, we encourage the development of a European collaborative framework, as proposed by the STAMP expert group, in which academic and not-for-profit organizations, pharmaceutical industry, health technology assessment bodies, payers, and regulators can work together on developing new uses for marketed medicines. Interestingly, EMA has expressed their support for the development and implementation of such a framework in their regulatory strategy for 2025 (Hines et al., 2019). The outcome of the planned pilot project to test the proposed repurposing framework will hopefully provide more insights on the outstanding issues for bringing new uses on-label and indicate potential next steps for the benefit of public health.

## Author Contributions

IH, IR, LM, and CV participated in the design of the study. CV reviewed the legislation and literature and drafted the initial version of the manuscript. IR, IH, and LM revised the manuscript critically and contributed to the interpretation of the data. All authors read and approved the final manuscript.

## Funding

CV's work as a PhD researcher at the KU Leuven was supported by a grant from the Anticancer Fund.

## Conflict of Interest

The authors declare that the research was conducted in the absence of any commercial or financial relationships that could be construed as a potential conflict of interest.
